# Two Novel Peptide Toxins from the Spider *Cyriopagopus longipes* Inhibit Tetrodotoxin-Sensitive Sodium Channels

**DOI:** 10.3390/toxins12090529

**Published:** 2020-08-19

**Authors:** Qingfeng Zhang, Yuxin Si, Li Yang, Li Wang, Shuijiao Peng, Yiming Chen, Minzhi Chen, Xi Zhou, Zhonghua Liu

**Affiliations:** The National and Local Joint Engineering Laboratory of Animal Peptide Drug Development, College of Life Sciences, Hunan Normal University, Changsha 410081, China; zhangqingfeng1224@163.com (Q.Z.); yuxinsi1996@163.com (Y.S.); yangli18737315273@163.com (L.Y.); wangli19966991@163.com (L.W.); psj15616053467@163.com (S.P.); chenyiming@smail.hunnu.edu.cn (Y.C.); chenmz@hunnu.edu.cn (M.C.)

**Keywords:** peptide toxins, sodium channel inhibitor, NaV1.7, site 4 neurotoxin

## Abstract

Sodium channels play a critical role in the generation and propagation of action potentials in excitable tissues, such as nerves, cardiac muscle, and skeletal muscle, and are the primary targets of toxins found in animal venoms. Here, two novel peptide toxins (Cl6a and Cl6b) were isolated from the venom of the spider *Cyriopagopus longipes* and characterized. Cl6a and Cl6b were shown to be inhibitors of tetrodotoxin-sensitive (TTX-S), but not TTX-resistant, sodium channels. Among the TTX-S channels investigated, Cl6a and Cl6b showed the highest degree of inhibition against NaV1.7 (half-maximal inhibitory concentration (IC_50_) of 11.0 ± 2.5 nM and 18.8 ± 2.4 nM, respectively) in an irreversible manner that does not alter channel activation, inactivation, or repriming kinetics. Moreover, analysis of NaV1.7/NaV1.8 chimeric channels revealed that Cl6b is a site 4 neurotoxin. Site-directed mutagenesis analysis indicated that D816, V817, and E818 observably affected the efficacy of the Cl6b-NaV1.7 interaction, suggesting that these residues might directly affect the interaction of NaV1.7 with Cl6b. Taken together, these two novel peptide toxins act as potent and sustained NaV1.7 blockers and may have potential in the pharmacological study of sodium channels.

## 1. Introduction

Voltage-gated sodium channels are essential for the initiation and propagation of action potentials that generate electrical impulses in neurons, cardiac muscle, and skeletal muscle, as well as in other excitable cells [1]. Sodium channels are composed of a pore-forming α-subunit and one or two auxiliary β-subunits [1,2]. The α-subunit can function independently, while β-subunits modulate gating kinetics and membrane localization of the α-subunit [3,4]. To date, nine sodium channel α-subunits (NaV1.1–1.9) and four sodium channel β-subunits (β1–β4) have been identified in mammals. Sodium channel α-subunits are composed of four homologous domains (DI–IV), each containing six hydrophobic transmembrane segments (S1–S6) [1,2]. The S5–S6 helices of each α-subunit domain contribute to pore channel formation, which is responsible for Na^+^ conduction across the membrane. The S1–S4 helices of each domain constitute the “voltage sensor”, which detects changes in membrane potential. Segment S4 contains multiple positively charged amino acid residues that respond to changes in membrane potential. After stimulation, segment S4 undergoes a conformational change that results in opening or closing of the sodium channel [5,6]. Sodium channel α-subunits are differently distributed among tissues: NaV1.1, 1.2, 1.3, and 1.6 are primary sodium channels in the central nervous system; NaV1.4 is mainly expressed in skeletal muscle; NaV1.5 is primarily expressed in cardiac muscle; NaV1.7, 1.8, and 1.9 are preferentially expressed in the peripheral terminals of sensory neurons [7,8,9]. Furthermore, sodium channel α-subunits are classified according to their sensitivity to tetrodotoxin (TTX): NaV1.5, NaV1.8, and Nav1.9 are TTX-resistant (TTX-R), whereas other α-subunits are sensitive to TTX (TTX-S) at nanomolar concentrations [2,10].

Mutations in sodium channels are associated with a variety of pathological disorders (e.g., epilepsy, pain, arrhythmia, etc.), and are important targets for the development of novel therapeutic agents [9,11]. In fact, currently marketed local anesthetic and antiepileptic drugs are largely sodium channel blockers [12]. Venomous animals (spiders, scorpions, centipedes, cone snails, etc.) evolved to produce toxins that can modulate the activity of sodium channels for predation or defense purposes [13,14,15,16]. Therefore, animal venom is a rich source of sodium channel modulators and potential therapeutic compounds. These specific modulators are not only powerful pharmacological tools that can be used to explore the physiological roles of sodium channels, but also serve as novel drug candidates and therapeutic targets [17,18,19,20,21,22,23,24]. In previous studies, several peptide toxins obtained from spider venoms were described to selectively target NaV1.1–NaV1.9 channels. Specifically, a few NaV1.7 channel-selective inhibitors were identified, such as Pn3a and HNTX-III, which belong to the spider-venom NaV toxins (NaSpTx) family and consist of 33–35 amino acid residues with three disulfide bridges that form a conserved inhibitor cystine knot (ICK) scaffold [25,26].

In this study, two novel 33-residue peptide toxins, namely μ-THTX-Cl6a (Cl6a) and μ-THTX-Cl6b (Cl6b), were isolated from the venom of the spider *Cyriopagopus longipes* and were shown to significantly reduce currents of the TTX-S channel with high affinity against NaV1.7. The mechanism underlying Cl6b activity on NaV1.7 was found to be similar to other previously reported NaV1.7-peptide inhibitors, i.e., by direct binding to the domain II segments S3–S4 (DII S3–S4) of NaV1.7 [5,19,25,27,28]. Cl6a and Cl6b showed strong binding kinetics which were linked to sustained NaV1.7 inhibition. Given the above mentioned characteristics of Cl6a/Cl6b–NaV1.7 interaction, the present findings may provide two valuable tools to study the pharmacological, structural, and functional features of NaV1.7.

## 2. Results

### 2.1. Purification and Characterization of Two Peptide Toxins (Cl6a and Cl6b) from Crude Venom of Spider C. longipes

**Figure 1:**
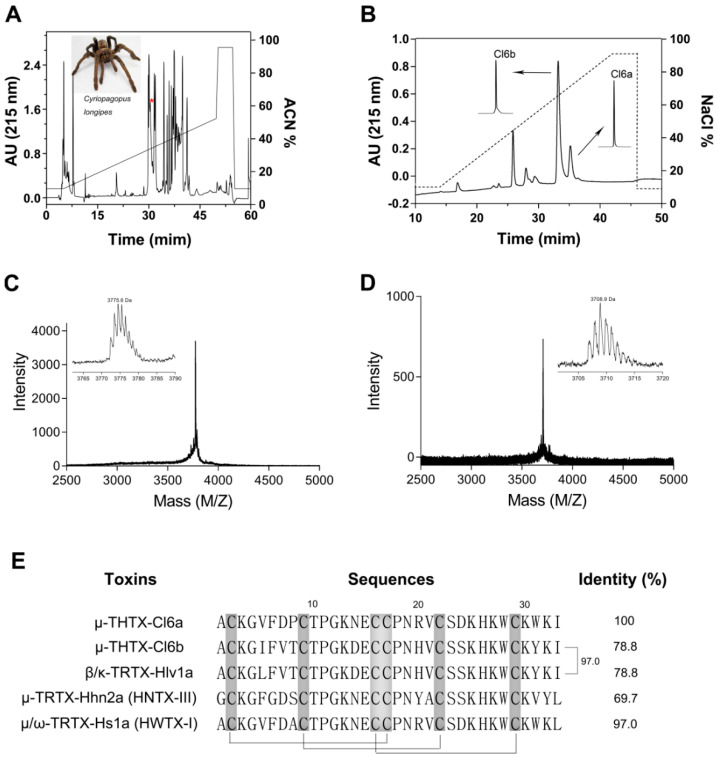
Extraction and purification of Cl6a and Cl6b peptide toxins from the venom of spider *Cyriopagopus longipes*. (**A**) Purification of spider venom through reverse-phase HPLC (RP-HPLC). The peak containing Cl6a and Cl6b fractions is indicated with a red asterisk. (**B**) Cl6a and Cl6b were purified to homogeneity by ion-exchange HPLC. The inserts show the desalting and further purification of the active fraction by RP-HPLC. (C, D) MALDI–TOF mass spectra of isolated Cl6a (**C**) and Cl6b (**D**), showing the monoisotopic mass of each toxin in daltons. (**E**) Sequence alignment of Cl6a and Cl6b against similar toxins. Cysteines shaded in gray form disulfide bonds and gray lines show the disulfide linkage.

Using semipreparative RP-HPLC, a total of 40 fractions were eluted from the crude venom extract of the spider *C. longipes* (Figure 1A). Further purification of recovered fractions by ion-exchange HPLC and analytical RP-HPLC yielded two active peptides (against NaV1.7), namely μ-THTX-Cl6a (Cl6a) and μ-THTX-Cl6b (Cl6b) (Figure 1B). The molecular weights of Cl6a and Cl6b were 3775.6 Da and 3708.9 Da, respectively (Figure 1 C,D). Next, amino acid sequencing was performed, showing that both Cl6a and Cl6b contained 33 amino acid residues with 78.8% sequence identity (Figure 1E). Six cysteine residues in Cl6a and Cl6b engaged to form three disulfide bonds. The purified toxins were found to share high sequence similarity with other previously characterized spider peptide toxins adopting an ICK motif, such as HNTX-III, HWTX-I, and GpTx1 [25,29,30]. Therefore, Cl6a and Cl6b were considered to adopt the same ICK scaffold with a similar cysteine pattern, thereby belonging to the NaSpTx family 1 (Figure 1E). Moreover, analysis of sequence alignment showed that Cl6a shared high identity (97%) with huwentoxin-I (HWTX-I), an inhibitor of N-type calcium channels and a TTX-sensitive sodium channel from the venomous spider *Ornithoctonus huwena*, and hainantoxin-III (HNTX-III) (67% identity), a selective inhibitor of NaV1.7 from the spider *Ornithoctonus hainana* [25,31,32]. Cl6b showed high similarity with beta/kappa-theraphotoxin-Hlv1a (97% identity) from the spider *Haplopelma lividum* (Figure 1E); this activity has not yet been determined.

### 2.2. Selectivity of Cl6a and Cl6b for Voltage-Gated Sodium Channel Subtypes

Next, the selectivity of Cl6a and Cl6b was investigated for other sodium channel subtypes expressed in HEK293T cells or ND7/23 cells. Inward currents in NaV1.2–NaV1.7 channels were evoked by a 50-ms depolarization potential of −10 mV from a holding potential of −100 mV. The depolarization potential for NaV1.8 was kept at +20 mV. The NaV1.9 current was evoked to −40 mV by a 50-ms depolarization potential from a holding potential of −120 mV. Furthermore, NaV1.8 and NaV1.9 currents were evoked in the presence of 1 μM TTX.

**Figure 2:**
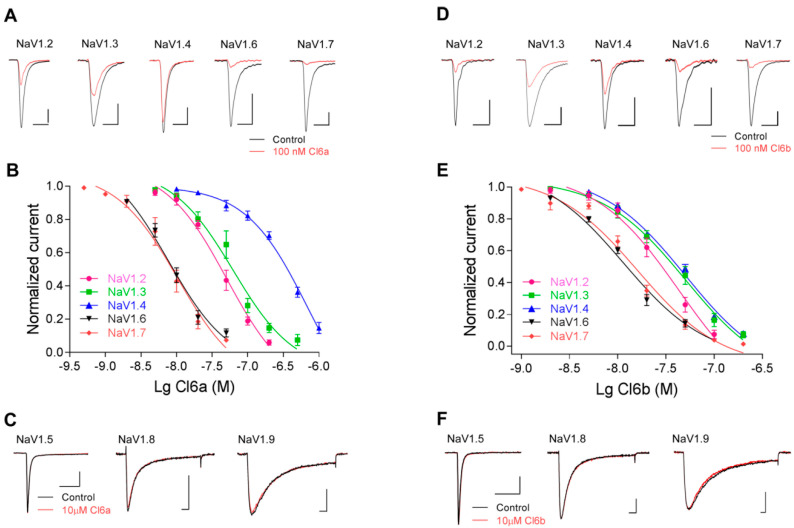
Effects of Cl6a and Cl6b on NaV1.2–1.9 sodium channels. (**A**,**B**) Representative current traces (**A**) and dose-dependent inhibitory curves (**B**) of Cl6a on NaV1.2–1.4, NaV1.6, and NaV1.7 a in whole-cell patch-clamp recordings. The black line indicates the control and the red line indicates the response after treatment with 100 nM Cl6a. Scale bar: 1 nA, 1 ms. (**C**) Treatment with Cl6a at a high concentration (10 µM) on NaV1.5, NaV1.8, and NaV1.9 channel currents. Scale bar: 1 nA, 5 ms. (**D**,**E**) Representative current traces (**D**) and dose-dependent inhibitory curves (**E**) of Cl6b on NaV1.2–1.4, NaV1.6, and NaV1.7 as assessed with whole-cell patch-clamp experiments. The black line indicates the control and the red line indicates the response after treatment with 100 nM Cl6b. Scale bar: 1 nA, 1 ms. (**F**) Treatment with Cl6b at a high concentration (10 µM) on NaV1.5, NaV1.8, and NaV1.9 channel currents. Scale bar: 1 nA, 5 ms. Data are presented as the mean ± standard error of the mean (SEM) (*n* = 4–6).

As shown in Figure 2A, 100 nM Cl6a completely inhibited hNaV1.7 and mNaV1.6 currents in HEK293T cells, and decreased rNaV1.2, rNaV1.3, and rNaV1.4 currents. In addition, the half-maximal inhibitory concentration (IC_50_) of Cl6a was determined to further evaluate its potency and selectivity profile. The highest potency was verified against hNaV1.7 (IC_50_ of 11.0 ± 2.5 nM) and mNaV1.6 (IC_50_ of 9.2 ± 1.3 nM), with progressively less potency against rNaV1.2 (IC_50_ of 56.1 ± 13.6 nM), rNaV1.3 (IC_50_ of 75.9 ± 6.3 nM), and rNaV1.4 (IC_50_ of 479.6 ± 100 nM) (Figure 2A,B and Table 1). No inhibitory effects were observed against hNaV1.5, rNaV1.8, or hNaV1.9 currents, even at concentrations of up to 10 µM (Figure 2C). Similarly to Cl6a, 100 nM Cl6b potentially inhibited the activity of rNaV1.2 (IC_50_ of 35.6 ± 7.3 nM), rNaV1.3 (IC_50_ of 61.6 ± 15.2 nM), rNaV1.4 (IC_50_ of 51.5 ± 6.9 nM), mNaV1.6 (IC_50_ of 12.6 ± 0.3 nM), and hNaV1.7 (IC_50_ of 18.8 ± 2.4 nM) (Figure 2D,E and Table 1), however, no effects were observed on hNaV1.5, rNaV1.8, or hNaV1.9 at 10 µM Cl6b (Figure 2F). Taken together, Cl6a and Cl6b share a similar affinity for NaV1.7 and NaV1.6 channels, whereas the selectivity for NaV1.4 varies dramatically between Cl6a and Cl6b, despite sharing a conserved core of amino acid residues. These findings indicate that Cl6a and Cl6b can inhibit TTX-S, but not TTX-R, sodium channels.

### 2.3. Effects of Cl6a and Cl6b on NaV1.7 Gating Kinetics

**Figure 3:**
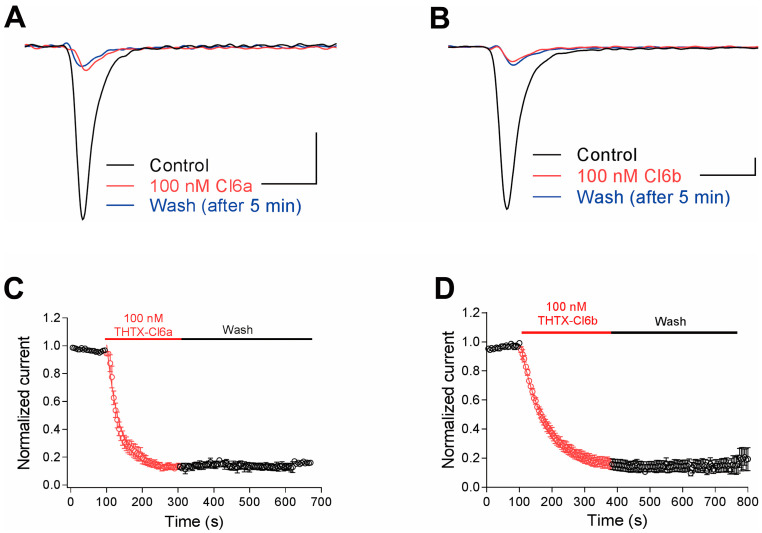
Cl6a and Cl6b irreversibly inhibit NaV1.7 channel currents. (**A**,**B**) Representative current traces from NaV1.7 in the absence (black) and presence (red) of 100 nM Cl6a and Cl6b. Blue lines represent currents recorded 5 min after washing with bath solution. Scale bar: 1 nA, 1 ms. (**C**,**D**) Time course of the block development induced by 100 nM Cl6a (C, *n* = 3) and Cl6b (D, *n* = 3) in peak currents of NaV1.7 channels, and recovery from inactivation after washing with bath solution. Data are presented as the mean ± SEM.

NaV1.7, NaV1.8, and NaV1.9 sodium channel subunits are known to play critical roles in the regulation of peripheral pain in mammals, thereby making them attractive therapeutic targets [11,19]. Cl6a and Cl6b showed strong inhibition against NaV1.7 but not against NaV1.8 and NaV1.9, indicating that these toxins might be potential candidates for pain treatment as well as for probing the pharmacology of NaV1.7. Hence, in this study, the effects of Cl6a and Cl6b on NaV1.7 gating kinetics were investigated. In time-dependent inhibition assays, Cl6a and Cl6b used at 100 nM inhibited a NaV1.7 peak current, and a slow onset of action was observed for both toxins (τ_on_ of 36.2 ± 5.3 s and 78.5 ± 14.7 s, respectively) (Figure 3A–D). Interestingly, Cl6a and Cl6b were washed off at an extremely slow rate from the NaV1.7 channel, as demonstrated by the inability of the NaV1.7 current to recover to control levels in 5 min (Figure 3A–D). Taken together, Cl6a and Cl6b irreversibly inhibit NaV1.7 channel currents, a characteristic that may be useful for the future development of NaV1.7 inhibitors with in vivo activity.

**Figure 4:**
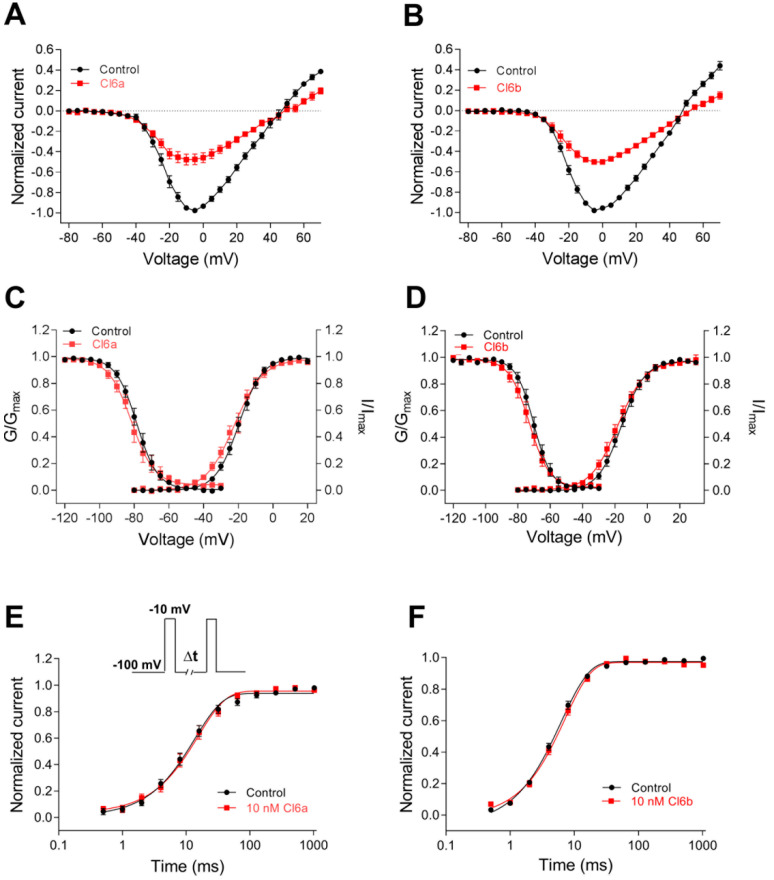
Kinetics of Cl6a and Cl6b interaction with NaV1.7 channels. (**A**,**B**) Current–voltage (I–V) relationships before and after treatment with 10 nM Cl6a (**A**, *n* = 10) and Cl6b (**B**, *n* = 10) at the NaV1.7 channel. (**C**,**D**) Curves of voltage-dependence steady-state activation and inactivation at the NaV1.7 channel before and after treatment with 10 nM Cl6a (**C**) and Cl6b (**D**), *n* = 6–10. (**E**,**F**) Kinetics of current recovery from fast inactivation of the NaV1.7 channel before and after treatment with 10 nM Cl6a (**E**, *n* = 9) and Cl6b (**F**, *n* = 7). Cells were prepulsed to −10 mV for 50 ms to inactivate NaV1.7 currents, then returned to the recovery potential (−100 mV) for increasing recovery durations prior (∆t) to a test pulse of −10 mV. The inset shows the pulse protocol for the recovery measurement from fast inactivation. Data are presented as the mean ± SEM.

Sodium channels can adopt the three distinct states of resting (closed), open, and inactivated. Channel gating refers to the movement of voltage sensors in response to changes in charge distribution across the cellular membrane, leading to rearrangement of the channel structure [2,6]. Most ICK-scaffold peptides are gating modifier toxins, which can alter kinetics and gating behavior of voltage-gated ion channels [24,33]. Thus, in this study, the effects of Cl6a and Cl6b on the kinetics of activation and inactivation of NaV1.7 channels were investigated. As shown in Figure 4A,B, Cl6a and Cl6b, when used at 10 nM, reduced NaV1.7 channel currents at –5 mV by 52.3% and 49.7%, respectively. The subsaturation concentration of 10 nM was subsequently used in gating kinetic assays. To measure current–voltage (I–V) relationships, potentials ranging from −100 mV to +70 mV in 5 mV increments were applied from a holding potential of −100 mV for 50 ms at 5 s intervals. As shown in Figure 4A,B, Cl6a and Cl6b decreased the current amplitude but did not alter the initial activation voltage, activation voltage of inward peak current, or reversal potential, suggesting that the toxin–NaV1.7 interaction did not determine change in ion selectivity. In addition, Cl6a and Cl6b showed no significant effect on voltage dependence of steady-state activation and inactivation of NaV1.7 channels (Figure 4CD, Table 2). Furthermore, Cl6a and Cl6b did not alter the repriming (recovery from inactivation) kinetics of NaV1.7 channels (Figure 4E,F, Table 2).

### 2.4. Cl6b Binds to the Domain II S3-S4 Linker of NaV1.7 Channels

**Figure 5:**
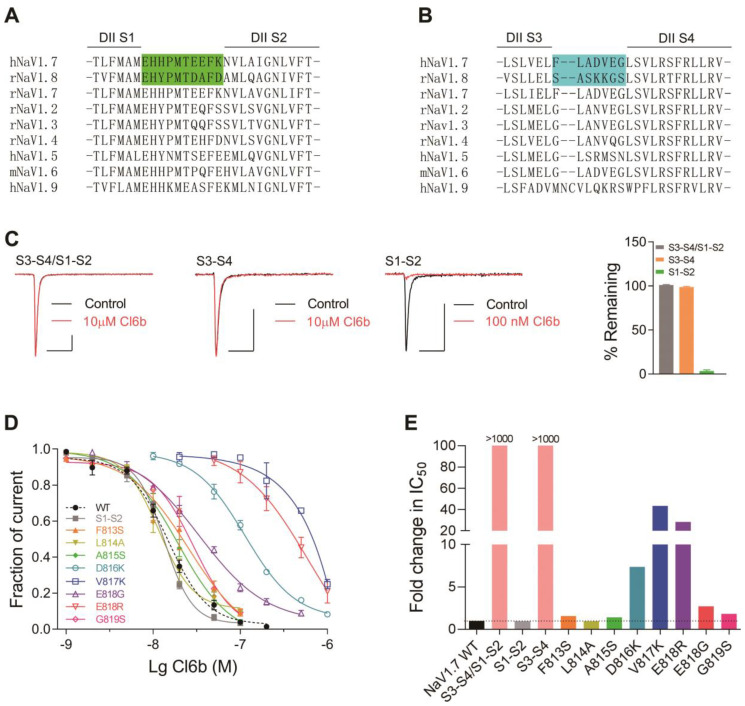
Effects of Cl6b on wild type (WT) and mutant hNaV1.7 channels expressed in HEK293T cells. (**A**,**B**) Sequence alignments corresponding to NaV subtype domains II (DII) S1–S2 (**A**) and DII S3–S4 (**B**). NaV1.7 and NaV1.8 nucleotide swaps via site-directed mutagenesis are highlighted. (**C**) Representative current traces from NaV1.7/1.8 DII S3–S4/S1–S2, NaV1.7/1.8 DII S3–S4, and NaV1.7/1.8 DII S1–S2 chimaera channels in the absence (black lines) or presence (red lines) of C16b. Scatter plot showing the remaining current after treatment with Cl6b (right). Scale bar: 1 nA, 5 ms. (**D**) Dose-dependent inhibitory curves of WT and mutant NaV1.7 channel currents after treatment with Cl6b. (**E**) Comparison between IC_50_ values of Cl6b for mutant and WT NaV1.7 channels. N = 4–7. Data are presented as the mean ± SEM.

Neurotoxins that act on voltage-gated sodium channels can target six different sites in channels, with site 4 being the hotspot for spider peptide toxins [33]. In this study, the underlying mechanism of action of Cl6a and Cl6b acting on NaV1.7 channels was similar to that described for HWTX-IV and HNTX-III, which are also site 4 toxins that interact with the DII S3–S4 linker of NaV1.7 channels [25,27]. We hypothesized that Cl6a and Cl6b would also inhibit NaV1.7 currents by targeting DII S3–S4. To identify the region of NaV1.7 critical for the toxin-induced inhibition of peak currents, several chimeric channels were constructed. NaV1.8 was resistant to Cl6a and Cl6b, therefore, three NaV1.7/NaV1.8 chimeric channels were constructed: NaV1.7/NaV1.8 DII S1–S2/S3–S4 (DII S1–S2/S3–S4), NaV1.7/NaV1.8 DII S1–S2 (DII S1–S2), and NaV1.7/NaV1.8 DII S3–S4 (DII S3–S4) (Figure 5A,B). Our findings revealed that substituting the extracellular region of NaV1.7 DII S1-S4 with the corresponding region of NaV1.8 (DII S1–S4 chimeric channel) caused the chimeric channel to be completely insensitive to Cl6b. In addition, Cl6b used at a high concentration (10 µM) had no effect on the DII S3–S4 chimeric channel, however, it significantly inhibited the current amplitude of the DII S1–S2 chimeric channel (Figure 5 Caken together, these results suggest that the DII S3–S4 linker of the NaV1.7 channel plays a key role in Cl6b-mediated inhibition. To elucidate the mechanism underlying Cl6b binding to hNaV1.7, amino acid residues in the S3–S4 linker region of hNaV1.7 (Figure 5B) were replaced by the corresponding amino acid residues of rNaV1.8 using site-directed mutagenesis. The results indicated that three component residues (D816, V817, and E818) were critical for Cl6b binding in the DII S3–S4 (Figure 5D). Furthermore, mutations in D816, V817, and E818 reduced the sensitivity to Cl6b by 7-fold, 43-fold, and 3-fold, respectively (Figure 5D,E). In NaV1.7, the residue D816 was shown to be conserved in NaV1.6 (corresponding to D834), while Gln residues were located in the corresponding positions of NaV1.2, NaV1.3, and NaV1.4 (Figure 5B). These results are consistent with the selectivity of Cl6b for TTX-S sodium channels (the IC_50_ value was NaV1.7 or NaV1.6 < NaV1.2, NaV1.3, or NaV1.4) (Figure 2E and Table 1). Interestingly, when the acidic residue Glu-818 was replaced with Lys, a basic amino acid, the channel sensitivity to Cl6b decreased 28-fold. Toxins of the NaSpTx family 1 contain conserved the motif (R/K)X(R/K)WCK (Cl6a and Cl6b contain KHKWCK), with the residues in this motif forming the positively charged surface [21,25,30,34,35]. In previous studies, this basic surface was indicated to directly interact with the acidic residues in site 4 to inhibit sodium channel currents [21,25,30]. This may also explain why no inhibitory activity of Cl6b on NaV1.5, NaV1.8, or NaV1.9 was observed, as these channels contain basic residues in site 4. Mutations in F813S, L814A, A815S, and G819S only slightly altered NaV1.7 sensitivity to Cl6b (Figure 5D,E). Based on these findings, the proposed mechanistic model involves Cl6b dockings at the DII S3–S4 linker of NaV1.7 channels mediated by the interaction with three amino acid residues (D816, V817, and E818).

## 3. Discussion and Conclusions

In this study, two peptide toxins (Cl6a and Cl6b) extracted from the venomous spider *C. longipes* were identified and characterized. Furthermore, the inhibitory effect of Cl6a and Cl6b was investigated in HEK293T and ND7/23 cells, which transiently express voltage-gated sodium channel subtypes. Cl6a and Cl6b inhibited TTX-S (NaV1.2–1.4, NaV1.6, and NaV1.7), but not TTX-R (NaV1.5, NaV1.8, and NaV1.9), sodium channels, suggesting that Cl6a and Cl6b are selective antagonists of TTX-S activity.

Sodium channels play an important role in the generation and propagation of electrical signals in excitable cells, often being the targets of toxins and drugs. Moreover, in genetic and functional studies, over 1000 mutations have been described in genes encoding nine sodium channel subtypes linked to a wide variety of human diseases [9]. Such genetic variants can offer a chance to better understand the mechanisms of related diseases and may constitute possible targets for the discovery of novel therapeutic drugs. For instance, loss-of-function mutations in *SCN9A*, which encodes NaV1.7, lead to congenital inability to experience pain (CIP) in humans [36,37]. This evidence prompted the notion that selective blockage of the NaV1.7 channel could relieve pain, which opened way to an industry-wide hunt for novel analgesics. Some spider venom-derived peptide toxins exhibit high affinity for NaV1.7 channels [33]. To this extent, Cl6a and Cl6b inhibited NaV1.7 channels with IC_50_ values of 11.0 nM and 18.8 nM, respectively. In addition, venom-derived peptide toxins display selectivity for sodium channel subtypes. For example, GpTx1 and Cl6a showed ~40-fold and 1000-fold inhibition over NaV1.4 and NaV1.5, respectively. However, engineered GpTx-1 analogues selectively inhibited NaV1.4 and NaV1.5 currents by 1000-fold [30]. Blocking NaV1.4 and NaV1.5 could be a major challenge in the development of NaV1.7 antagonists, because this blockage might impair normal function of skeletal and cardiac muscle and lead to dyspnea and arrhythmia, possibly even culminating in death [38,39]. Moreover, most peptide toxins (including Cl6a and Cl6b) from venomous animals conform to the ICK motif (Figure 1C), which confers chemical, thermal, and biological stability [40,41]. Therefore, venom-derived peptide toxins may be a valuable resource for the development of NaV1.7 drugs.

To date, more than 30 peptide toxins derived from spider venoms with activity against NaV1.7 have been identified. Based on the primary sequences and disulfide scaffolds, these toxins belong to NaSpTx families 1–3, which bind to site 4 (DII S3-S4) to depress channel activity currents using different mechanisms of action [21,33]. NaSpTx2 and NaSpTx3 toxins shift the voltage dependence of activation in the positive direction [33]. Conversely, this work alongside previous studies indicated that NaSpTx1 (e.g., HNTX-III and GpTx-1, including Cl6a and Cl6b) cannot significantly change the activation kinetics of sodium channels. However, when compared to HNTX-III and GpTx1, the NaV1.7-inhibiting properties of Cl6a and Cl6b are distinct; whereas HNTX-III and GpTx1 cause NaV1.7 inhibition that is reversible upon washing, Cl6a and Cl6b induce a sustained inhibition of NaV1.7 channel currents (Figure 3D). Such characteristics could be useful for prolonging the blockage of NaV1.7 activity, which may be beneficial for long-term analgesia in vivo. Therefore, Cl6a and Cl6b may serve as a base framework upon which novel drugs targeting NaV1.7 could be developed, although they have poor selectivity in sodium subtypes.

## 4. Materials and Methods

### 4.1. Venom and Peptide Toxin Purification

The crude venom of *C. longipes* was obtained by electrical stimulation, freeze-dried, and stored at −80 °C until further use. Lyophilized venom was dissolved in double-distilled water (ddH_2_O) and purified by both reverse-phase (RP-HPLC) and ion-exchange chromatography. RP-HPLC was performed using a C18 column (10 × 250 mm, 5 μm; Welch Materials Inc., Shanghai, China) on an analytical Waters 2795 HPLC system. The acetonitrile gradient increased at a rate of 1% per minute from 5–50%, using a flow rate of 3 mL/min. Eluted fractions were lyophilized and further fractionated using ion-exchange chromatography with an XB-SCX column (4.6 mm × 250 mm, 5 μm; Welch, China) on a preparative Hanbon HPLC system. The NaCl gradient increased at a rate of 2% per minute from 0–70% at a flow rate of 1 mL/min. Collected fractions containing Cl6a and Cl6b were subjected to further desalination by RP-HPLC using a C18 column (4.6 mm × 250 mm, 5 μm; Welch, China) on an analytical Waters 2795 HPLC system. In the second round of RP-HPLC, the acetonitrile gradient increased from 27–33% at a rate of 0.5% per minute and a flow rate of 1 mL/min. For C18 RP-HPLC, solvent A was 0.1% trifluoroacetic acid (TFA) in water and solvent B was 0.1% TFA in acetonitrile. Cl6a- and Cl6b-containing fractions were lyophilized and stored at −20 °C until further use.

### 4.2. Mass Spectrometry Analysis and Amino Acid Sequencing

The purity and molecular weights of Cl6a and Cl6b were determined by matrix-assisted laser desorption/ionization–timeof-flight mass spectroscopy (MALDI-TOF MS) analysis in an AB SCIEX TOF/TOF™ 5800 system (Applied Biosystems, CA, USA). The entire amino acid sequences of both peptides were obtained by automated Edman degradation in a PPSQ-53A protein sequencer (Shimadzu Corporation, Kyoto, Japan)

### 4.3. Plasmids and Transfection Experiments

Human NaV1.5 and NaV1.7, rat NaV1.2, NaV1.3, NaV1.4, and NaV1.8, and mouse NaV1.6 clones and beta subunit (β1 and β2) clones were donated by Dr. Theodore R. Cummins (Department of Pharmacology and Toxicology, Indiana University School of Medicine, USA). Plasmid vectors used for the subcloning of hNaV1.7 and rNaV1.8 were pcDNA3.1 and pCMV-blank, respectively. Human NaV1.9 (hNaV1.9) was synthesized (Genscript) and subcloned into the pEGFP-N1 vector. The C-terminal of hNaV1.9 was linked to a green fluorescent protein (GFP) to yield a fusion protein channel (hNaV1.9-GFP) [41]. Mutations in hNaV1.7 were generated using the GeneTailor™ Site-Directed Mutagenesis system (Thermo Fisher Scientific), according to the manufacturer’s guidelines. NaV1.2–NaV1.7 and mutant plasmids containing engineered GFP (eGFP) were transiently transfected into HEK293T cells using Lipofectamine 2000 (Invitrogen, Carlsbad, CA, USA). Additionally, plasmids β1- and β2-eGFP encoding human β1 and β2 subunits, respectively, were co-transfected with those encoding WT NaV1.7 and mutated NaV1.7 into HEK293T cells. NaV1.8 was co-transfected with eGFP and transiently transfected into ND7/23 cells. hNaV1.9-eGFP was transfected into ND7/23 cells as previously described [42]. HEK293T and ND7/23 cells were obtained from the Shanghai Institute of Cell Biology, Chinese Academy of Sciences (Shanghai, China), and maintained with 5% CO_2_ at 37 °C in Dulbecco’s Modified Eagle’s Medium (DMEM) supplemented with 10% fetal bovine serum (FBS), penicillin (100 U/mL), and streptomycin (100 µg/mL). At 24 h after transfection, cells exhibiting GFP fluorescence were used in whole-cell patch-clamp recordings.

### 4.4. Electrophysiology Recordings

Whole-cell patch-clamp recordings were performed at 25 ± 2 °C in an EPC 10 USB patch clamp amplifier (HEKA Elektronik, Lambrecht, Germany). Recording pipettes with access resistance of 2.0–3.0 MΩ were fabricated from borosilicate glass capillary tubes using a two-step vertical microelectrode PC-10 puller (Narishige Group, Tokyo, Japan). Voltage-clamp recordings were acquired with PatchMaster software v2 × 73 (HEKA Elektronik) 5 min after establishing whole-cell configuration, sampled at 20 kHz, and filtered at 5 kHz. Voltage errors were minimized with 80% series resistance compensation. Pipettes were filled with (mM) 105 CsF, 35 NaCl, 1 EGTA, and 10 HEPES (pH 7.4, adjusted with CsOH). The external solution contained (mM) 140 NaCl, 2 CaCl_2_, 1 MgCl_2_, 2 KCl, 10 HEPES, and 10 glucose (pH 7.4, adjusted with NaOH).

### 4.5. Data Analysis

Data analysis was performed with Igor Pro 6 (WaveMetrics, Lake Oswego, OR, USA), GraphPad Prism 7 (GraphPad Software Inc., CA, USA), and Office Excel 2010 (Microsoft Corporation, WA, USA). All values are shown as mean ± standard error of the mean (SEM) with *n* representing the number of examined cells. Significant levels were set at *p* < 0.05. Statistical analyses were performed with Prism 7 (Version 7.00, GraphPad) software.

## Figures and Tables

**Table 1:** Half-maximal inhibitory concentration (IC_50_) value of several sodium channel subtypes by Cl6a and Cl6b.

Sodium Channel Subtypes	Cl6a (nM)	Cl6b (nM)
rNaV1.2	56.1 ± 13.6	35.6 ± 7.3
rNaV1.3	75.9 ± 6.3	61.6 ± 15.2
rNaV1.4	479.6 ± 100	51.5 ± 6.9
mNaV1.6	9.2 ± 1.3	12.6 ± 0.3
hNaV1.7	11.0 ± 2.5	18.8 ± 2.4

**Table 2:** The effects of 10 nM Cl6a and Cl6b on voltage dependence of activation and inactivation and repriming of the NaV1.7 channel.

	Control					Toxin				
	Voltage Dependence of Activation (mV)		Voltage Dependence of Inactivation (mV)		Repriming (ms)	Voltage Dependence of Activation (mV)		Voltage Dependence of Inactivation (mV)		Repriming (ms)
	*V*_1/2_	k	*V*_1/2_	k		*V*_1/2_	k	*V*_1/2_	k	
**Cl6a**	−19.5 ± 1.5	6.2 ± 0.3	−78.3 ± 1.8	−5.4 ± 0.3	14.9 ± 2.4	−22.3 ± 1.7	7.3 ± 0.4	−81.6 ± 2.4	−5.7 ± 0.4	17.6 ± 2.5
**Cl6b**	−16.3 ± 0.4	6.7 ± 0.4	−70.1 ± 1.5	−5.2 ± 0.3	6.3 ± 0.9	−19.3 ± 1.7	7.0 ± 0.5	−73.1 ± 1.8	−5.7 ± 0.4	7.1 ± 0.5
